# The Folk Sociological Imagination: Manufacturing Agency Through Smoking Among Chinese Adolescents

**DOI:** 10.1111/1467-9566.70173

**Published:** 2026-03-21

**Authors:** Bo Li

**Affiliations:** ^1^ Department of Applied Social Sciences The Hong Kong Polytechnic University Hong Kong Hong Kong

**Keywords:** adolescent smoking, agency, lay epidemiology, nicotine control, sense‐making, sociological imagination

## Abstract

Persistent adolescent smoking in China presents a paradox within the context of advancing nicotine control. Moving beyond social–environmental explanations, this study employs Mills' sociological imagination to conceptualise this persistence as an agentive response to constrained realities and futures, enacted through peer‐curated lay epidemiology. Its core argument is that adolescents cultivate a folk sociological imagination—a vernacular system of sense‐making—to manufacture agency and reframe smoking risk. Qualitative data from 21 adolescent smokers in Shenzhen, including 208 health diaries and 17 interviews, reveal how this is achieved through three practices: the selective valorisation of healthy smoker exemplars; folk attribution of causality to external or individual factors; and prevalence‐as‐safety normalisation. This folk process reconfigured the public issue of smoking risk into a series of manageable private troubles, transforming statistical harm into a matter of individual circumstance. Findings highlight three gaps in current efforts: an epistemic gap in policy, which dismisses peer‐validated evidence; an intervention gap in health education, which fails to engage with lay reasoning and a structural hope gap, which generates a form of cruel optimism that overlooks the need for alternative avenues for agency and belonging.

## 1 Introduction

**FIGURE 1:**
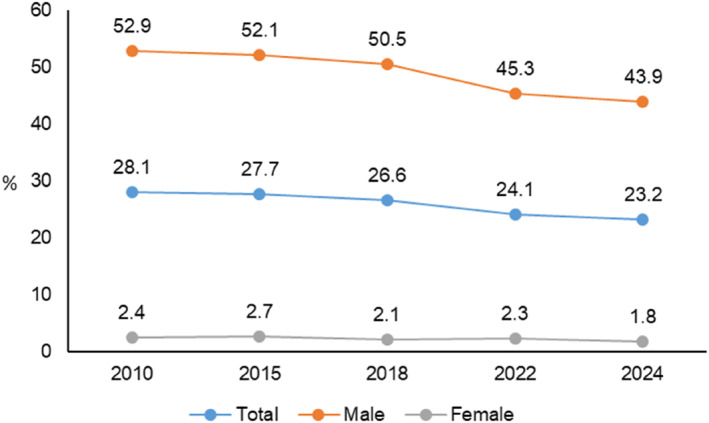
Smoking prevalence in China. (1) Survey population aged ≥ 15; (2) for adolescents (15–24 years), the current smoking prevalence is 10.7% (male: 18.7%; female: 1.7%). *Source:* 2024 China adult smoking survey (Chinese Center for Disease Contro and Prevention 2025).

Over the past 2 decades, a global decline in adolescent smoking stands in contrast to its persistent prevalence within China (Figure 1). Despite a progressive regulatory landscape, an estimated nine million Chinese adolescents aged 15–19 are regular smokers, supplemented by a further 18 million experimenters (Tencent News 2024). This entrenched behaviour amidst strengthening control measures constitutes a critical puzzle: why does adolescent smoking persist when prevailing explanatory models, focused on external determinants, fail to fully unravel?

In China, cigarettes are widely sold in convenience stores, whereas e‐cigarettes are available through pervasive dedicated retailers (e.g., Relx/悦刻). Adolescents may also informally obtain products from parents who smoke. Regulatory measures to curb access, though progressive in scope, encounter enforcement challenges. National legislation, including the Minors Protection Law and Tobacco Monopoly Law, prohibits sales to minors and requires age verification, with the E‐cigarette management measures imposing parallel restrictions (Ministry of Justice 2020; State Council 2022). Sales are also banned within 50–100 m of schools, and smoking is prohibited on school grounds (Ministry of Justice 2020). Parents, guardians, and schools bear a legal duty to discourage adolescent smoking (Supreme People’s and Procuratorate 2020). Yet enforcement is lax: parental smoking at school gates is commonly observed without sanction; jurisdictional ambiguity, especially concerning e‐cigarettes, further fragments oversight and public health messaging is inadequately tailored to adolescents' social worlds and ineffective against peer‐circulated narratives framing smoking as helpful (Law Index 2024). These gaps between policy and praxis sustain an environment in which nicotine products remain accessible.

Conventional sociological and public health understandings are dominated by a structuralist perspective, emphasising how adolescents are influenced by external social–environmental forces. This can be categorised into two strands: the regulatory–institutional and the socio‐cultural. The first strand, drawing from policy studies and social ecology models, highlights a gap between policy and praxis. It details how comprehensive legislation, from sales prohibitions to advertising bans, is undermined by inconsistent enforcement and commercial exploitation, evidenced by ‘ubiquitous’ point‐of‐sale marketing near schools and porous digital restrictions (Barker et al. 2024; Pettigrew et al. 2023; Wang et al. 2020; Zeng et al. 2025). The second strand, rooted in social learning and normative influence theories, emphasises the socio‐cultural dimensions. It positions peer networks as primary sites for socialisation, where smoking is adopted for acceptance and conformity, and frames the family and school as institutional environments where parental and teacher modelling normalise nicotine use, reinforcing its perception as an aspirational behaviour within a broader cultural repertoire (Cheng et al. 2022; Liu et al. 2017; Liu et al. 2025; Zhao et al. 2018).

Whereas these social‐environmental explanations provide a crucial mapping of the objective ‘fields’ of behaviour, their structuralist lens constitutes a significant analytical limitation. By framing adolescents primarily as passive subjects or cultural dupes who internalise external norms, it diminishes their capacity for agency (i.e., their ability to act upon and interpret their social world). This perspective, echoing a classic sociological tension between structure and agency (Guy 2019), elides the subjective, meaningful processes through which young people actively negotiate, contest and legitimise smoking practices. The central, unanswered question is not just how social forces (dis)enable smoking, but how adolescents themselves rationalise and sustain it against a backdrop of seemingly incontrovertible institutional evidence, thereby exercising a form of discursive agency.

It is this lacuna that this study addresses by introducing a theoretical reorientation. It proposes shifting from an over‐reliance on deterministic models towards a framework that centres the agentive, sense‐making social actor. Drawing upon Mills's sociological imagination, the study conceptualises adolescent smoking not as a mere failure of regulation or a product of influence, but as an agentive response to structurally constrained realities and futures. It contends that adolescents cultivate a *folk sociological imagination* through peer‐curated lay epidemiology, the process by which individuals interpret health risks through personal experience and social observation (Davison et al. 1991; Pihl et al. 2017). This constitutes a system of sense‐making that manufactures agency, belonging and a form of hope—understood through Berlant's (2011) concept of cruel optimism (an attachment to a harmful practice that sustains a sense of possibility in an untenable present)—amidst intense socio‐academic pressures, representing a pragmatic, if misguided, tool for reconfiguring the public issue of smoking risk into a manageable private trouble.

Accordingly, this study investigates how Chinese adolescent smokers construct and legitimise these alternative evidence systems. Through an analysis of health diaries and interviews, the study elucidates three practices underpinning this folk epistemology: the selective valorisation of healthy smoker exemplars; folk attribution of causality to external or individual factors and the normalisation of safety through perceived prevalence. Findings underscore the necessity for interventions that move beyond correcting misinformation to address the very deficit of legitimate agency, challenging us to create social structures that supplant cruel optimism with sustainable forms of hope without nicotine use.

Before proceeding, a brief note on terminology is warranted. This investigation examines the use of both combustible cigarettes and e‐cigarettes (commonly referred to as vaping). Although smoking technically denotes combustion and thus applies to traditional cigarettes, the term is employed throughout this paper as an umbrella descriptor for two interrelated reasons. First, all participants in this study were poly‐users who alternated between products according to context and availability, treating them as functionally interchangeable within their social repertoire. Second, and more fundamentally, the study's theoretical focus is on the social meanings and agentive reasoning that underpin nicotine use, rather than on a comparative health or behavioural analysis of different delivery mechanisms. For the purpose of examining how adolescents cultivate a folk sociological imagination, both products serve as culturally available resources for managing stress, performing identity, and forging belonging (Chen et al. 2022; Hua et al. 2023; Xie and Zhang 2025; Yang et al. 2025). Accordingly, the term ‘smoking’ is deployed heuristically to encompass this broader category of nicotine practices, a usage that faithfully reflects both participants' own vernacular and the practical interchangeability of these products in their everyday lives.

## 2 Theoretical Framework

This study is framed by an integrative theoretical perspective, combining Mills's (2000) sociological imagination with the theory of lay epidemiology to explain how adolescents interpret and legitimise health risks within their social worlds. It positions Mills's theory as a macro‐sociological foundation for understanding smoking as a response to biographically experienced constraints within historically shaped structures. Lay epidemiology serves as the explanatory micro‐sociological mechanism, connecting this macro lens to empirically observable sense‐making processes.

Mills's sociological imagination posits that the task of social science is to understand the intersection between biography and history, or private troubles and public issues. The immense academic pressures, competitive futures, and social constraints facing Chinese adolescents constitute the public issues, namely, the macro‐structural forces shaping their lives (Hao et al. 2024; Hou 2024). Their concomitant private troubles, such as stress, anxiety, and a search for identity and belonging, are experienced within this context (Chen et al. 2022; Fan and Li 2025; Hao et al. 2024; Hua et al. 2023; Yang et al. 2025). So, the sociological imagination is itself a form of agency: a capacity to recognise these forces and potentially act upon them (Mills 2000). When legitimate avenues for such agency are perceived as blocked, individuals may develop alternative, symbolic strategies to manufacture a sense of control. This study argues that smoking is one such strategy: a personalised solution to public problems.

To understand the specific mechanisms of this process, the study uses the theory of lay epidemiology. First articulated by Davison et al. (1991), lay epidemiology refers to the processes by which individuals and communities interpret health risks through personal experience, social observation and everyday reasoning, privileging this vernacular knowledge over official statistical evidence. It is a form of lay knowledge derived from the broader social, cultural and political contexts of lived experience (Lovatt et al. 2015). Its core premises that risk is socially constructed through lived experience and that observable cases outweigh probabilistic data illuminate how health beliefs are formed outside clinical settings (Nuti and Armstrong 2021). This constructivist approach contrasts with standard epidemiology by focusing on how risks are perceived and weighed within the holistic context of individuals' lives, values and social networks (Davison et al. 1991; Lovatt et al. 2015; Pihl et al. 2017).

This study integrates these two theories by considering lay epidemiology as the practical mechanism through which a folk sociological imagination is enacted. Within peer networks, adolescents utilise the tools of lay reasoning, including anecdotal evidence, causal storytelling and normative validation, to reconstruct the narrative around smoking. They reframe it from an incontrovertible public health issue into a manageable personal choice, thereby manufacturing the agency and a form of hope—what Berlant (2011) terms cruel optimism, wherein an attachment to a practice (e.g., smoking) is maintained because it provides immediate relief and social belonging, despite its long‐term harm—that the broader structure fails to provide. Their shared reasoning is thus not merely a cognitive bias but an agentive sense‐making practice.

This study conceptualises this process, whereby adolescents use lay epidemiological reasoning to manufacture agency amidst structural constraints, as the folk sociological imagination. This construct denotes a vernacular collective system of sense‐making that somewhat inverts the trajectory of Mills's sociological imagination. Where Mills's theory connects private troubles to public issues, the folk sociological imagination functions as a pragmatic, agentive tool through which peer networks reconstitute public issues as private, manageable troubles. It is not an absence of sociological thought but a socially sustained recalibration of it. This is apt for analysis because it focuses on the discursive and epistemic work through which agentive positions are constructed, namely, the peer‐curated production of alternative evidence, causal narratives and norms. While theories of practice (Bourdieu 2018) foreground embodied habitus within social fields, and theories of counterpublics (Warner 2005) address organised oppositional identity, folk sociological imagination illuminates the informal vernacular reasoning that reconfigures health risk in everyday adolescent life, without necessarily forming a counterpublic or expressing a durable disposition. It thus provides a fitting conceptual lens for understanding how institutional health messages are neutralised through collective sense‐making.

Guided by this integrated framework, this study investigates how Chinese adolescent smokers utilise lay epidemiological reasoning to construct a folk sociological imagination that legitimises smoking as an agentive response to structural constraint, addressing the following questions: (1) How do adolescents utilise personal and peer observations to construct alternative evidence systems that challenge institutional health messages?; (2) through what discursive strategies do they attribute causality to dissociate smoking from harm?; (3) how does the perceived prevalence of smoking within their social world function as a form of normative validation? and (4) in what ways do these processes constitute a folk sociological imagination that reconfigures the public issue of smoking risk into a manageable private trouble?

This framework guides analysis by focusing the inquiry on the narratives, discourses, and sense‐making processes evident in the data, interpreting them for their social function as strategies for building identity and asserting control. This theoretical approach moves beyond deterministic models, providing a bottom‐up agency‐focused account that explains why smoking persists not despite social‐environmental factors but because of the agentive ways adolescents ‘creatively’ respond to them through folk reasoning.

## 3 Methods

### 3.1 Design

A qualitative research design was employed as it prioritises depth, context and participant‐led meaning, aligning with the theoretical commitment to understanding how adolescents exercise agency rather than merely measuring behaviour or attitudes.

### 3.2 Setting

Shenzhen was selected as the study site due to its reputation for advanced nicotine control, which creates a revealing paradox central to this investigation. The city's Smoking Control Regulation, which prohibits sales within 50 m of schools, alongside initiatives like the ‘family–school–health’ coordination model (Shenzhen Municipal Government 2019; Shenzhen News 2024), represents a strong regulatory environment. Consequently, the city's adolescent smoking rate has been reduced to 1.1%, far below the national average of 10.7% (Chinese Center for Disease Contro and l and Prevention 2025; Public Hygiene and Health Commission of Shenzhen Municipality 2024).

It is this context of regulatory ‘success’ that makes Shenzhen a pertinent site for this study. The persistence of adolescent smoking (even at low levels) amidst such robust controls, coupled with high awareness of banned products such as e‐cigarettes and their availability in informal markets (Public Hygiene and Health Commission of Shenzhen Municipality 2024; The Paper 2019), illuminates the limitations of a purely structuralist explanation. This setting allows for a focused examination of how adolescents negotiate and find agency within a restrictive environment.

### 3.3 Participants

Twenty‐one adolescent smokers (aged 16–18; 19 males and 2 females) from three senior high schools in Shenzhen were recruited via snowball sampling. The sample's gender composition reflects the epidemiological pattern of higher smoking prevalence among males in China (Chinese Center for Disease Control and Prevention 2025), whereas also indicating the potential greater social visibility and network accessibility of male smoking peer groups. This method was selected not merely for convenience, but for its strong theoretical and phenomenological congruence with the study's focus on peer‐curated knowledge and communal sense‐making.

Given the sensitive and often concealed nature of adolescent smoking within a restrictive regulatory environment, this population is difficult to access via random or other purposive sampling methods. Snowball sampling is empirically established as an effective strategy for recruiting such ‘hidden/hard‐to‐reach populations’ by leveraging the trust and existing social networks of participants themselves (Sadler et al. 2010). This is a deliberate strategic choice: for a study investigating how health beliefs are circulated and validated within peer networks, the sampling method must mirror the very social phenomenon under scrutiny. The resulting sample is therefore not intended to be statistically representative but is ecologically valid, representing a real‐world interconnected social group within which the processes of lay epidemiological reasoning naturally occur.

Initial three participants were identified through my informal contacts, after which the sample expanded via peer referral (each referred one peer smoker but not all nominated others). This ensured access to networks of peers who engage in discussions about smoking, thereby facilitating the investigation of how beliefs are constructed and legitimised within these intimate social circles. It allowed the research to tap into the authentic social contexts where the folk sociological imagination is enacted.

Participants included users of both traditional cigarettes and e‐cigarettes to capture the full spectrum of current adolescent nicotine practices. As noted above, all participants were poly‐users who treated these products as functionally interchangeable within their social repertoire; the analysis therefore combines them to focus on the social meanings underpinning nicotine use rather than product‐specific comparisons.

### 3.4 Data Collection

Data were collected between May and July 2025, using two complementary methods.

#### 3.4.1 Health Diaries

Participants produced 208 diary entries (7–11 per participant, 78–109 words each). The diary method was chosen for its capacity to capture rich contemporaneous accounts of everyday reasoning in vivo (Akinreni et al. 2024; Stephens et al. 2025). To ground entries in the study's theoretical focus, participants were given the following guidance: ‘Please use this diary as a personal log. Each time you hear, observe, or take part in a conversation with friends or peers about smoking or vaping, especially about whether it is harmful, why some people get sick and others don't, or why people do it, jot down a brief entry. I am interested in your world and your conversations. Please try to capture: (1) What was the specific situation?; (2) What was said? (What reasons or examples did people give?); (3) Did anyone disagree? How was that handled? and (4) What did you think about the conversation afterwards?’ This guidance was designed to prompt documentation of the core processes of lay epidemiology (e.g., causal attribution) and peer validation as they occurred in everyday life.

#### 3.4.2 Interviews

Seventeen participants took part in semi‐structured interviews (43–68 min) (Adams 2015), conducted in Mandarin in neutral settings (cafés) to foster openness. The interviews served to triangulate and elaborate on the diary data, exploring how the interpretations documented therein were socially circulated, validated, or challenged within peer networks.

The interview protocol was structured around core themes derived from the theoretical framework, using the participants' own diary entries as a primary prompt to ensure participant‐led meaning remained central. Key prompts included the following: ‘In your diary, you mentioned a conversation about [for example, a healthy smoker]. Can you tell me more about that person? Why do you think they are brought up in these discussions?’, a question designed to probe the selective valorisation of exemplars. Others included, ‘You noted that someone said illness from smoking is due to [for example, “weak lungs” or “bad luck”]. How do you and your friends decide what really causes health problems? Where do these ideas come from?’ to explore folk causal attribution; and ‘When you see many people smoking, what does that mean to you and your friends? Does it make you think about the risks differently?’ to investigate prevalence‐as‐safety normalisation. Further prompts elicited lay epidemiological rhetoric (‘If you were to explain to a younger student why smoking isn't as dangerous as official messages say, what stories or examples would you use?’) and epistemic validation within peer networks (‘How do you know if a belief about smoking is true?’). These prompts were designed to probe the informal mechanisms, such as anecdotal comparisons, peer exemplars and local credibility cues, through which smoking‐related beliefs gain social legitimacy.

The interviews were audio‐recorded and transcribed verbatim. The analysis, including coding and theme development, was conducted directly on the Mandarin transcripts to preserve linguistic and cultural nuance. This in‐language analysis was essential for sensitively interpreting vernacular expressions and peer‐specific phrases, grounding findings in their immediate socio‐cultural context. Following the identification of stable themes and illustrative data excerpts, the relevant quotations and analytical summaries were translated into English for presentation. Translation employed a meaning‐based approach, prioritising conceptual equivalence to retain participants' original meanings, speech styles and cultural references [e.g., terms such as ‘身体好’ (healthy)]. This ensured analytical interpretations remained grounded in the participants' linguistic context, mitigating potential nuance loss.

Both diary entries and interviews, with their exploratory prompts connecting personal observations to broader discourses of risk and authority, were designed to move beyond description to elicit reflective, situated talk. They encouraged participants to articulate and scrutinise the lay epidemiological reasoning that typically remains tacit in everyday peer conversation.

Data collection and preliminary analysis occurred concurrently, allowing emerging themes to inform subsequent interviews in an iterative process until saturation was achieved (i.e., no new interpretive patterns or lay epidemiological processes were observed).

### 3.5 Data Analysis

Data were analysed using an abductive approach (Timmermans and Tavory 2022). Abduction is a mode of reasoning suited to generating theoretical insights from empirical puzzles. It involves a systematic, iterative dialogue between pre‐existing theoretical frameworks and emergent, surprising patterns in the data to develop the most plausible explanatory account (Timmermans and Tavory 2022). This approach was epistemologically appropriate for the study, as it embraces the complex socially constructed nature of lay health reasoning and facilitates the development of ‘practically relevant theoretical insight’ (Hulst and Visse 2025) from participants' everyday sense‐making practices. Specifically, it enabled the development of the folk sociological imagination by maintaining a creative tension between the guiding theories (i.e., Mills's sociological imagination, lay epidemiology) and the participant‐driven meanings that somewhat reworked them.

The entire dataset, comprising 208 diary entries and 17 interview transcripts, was prepared as a single corpus for analysis. The unit of analysis was any meaningful expression of health reasoning, ranging from a sentence to a paragraph. In total, approximately 2800 such units were identified and extracted from data for coding. The analytical process was managed using ATLAS.ti, while theme development was conducted manually to preserve nuanced contextual understanding.

The analysis began with repeated immersion in the texts for familiarisation. Initial coding of the 2800 units was conducted with theoretical sensitivity (Timmermans and Tavory 2022). Codes were informed by terms such as ‘agency’ and ‘peer validation’ but remained open to unexpected patterns. This resulted in descriptive codes capturing the substance of participants' lay reasoning (e.g., ‘grandfather smoked for 60 years healthy’, ‘blaming cough on air pollution’).

Initial codes were reviewed, compared across cases and data types, and grouped into candidate themes. This involved organising codes that spoke to a shared underlying pattern, moving from description to interpretation. Throughout, I maintained a reflective journal, writing memos to critique emerging themes and interrogate my role in knowledge construction. Themes were refined iteratively to ensure they accurately reflected the shared social processes of sense‐making central to the folk sociological imagination, rather than just cataloguing individual beliefs. This abductive process, continually moving from data to theory and back, was essential for conceptualising how the practices of selective exemplification, causal attribution and prevalence normalisation collectively constitute the folk sociological imagination.

### 3.6 Reflexivity

For adolescents navigating the intense pressures of high school in Shenzhen, my status as an external non‐judgemental researcher may have provided a rare and valued opportunity to confide in a neutral outsider. The research encounter itself might have become a sanctioned space to reflect upon the shared reasoning behind their smoking practices in a more systematic way than occurs in everyday peer exchange, inviting participants to articulate, defend and connect their beliefs to wider discourses of risk and authority.

Whereas this external status likely facilitated more open disclosures from participants, it also presented challenges. My theoretical commitment to understanding adolescent agency, although a strength of the study's design, required continual self‐vigilance to ensure that the desire to ‘find’ agentive meaning did not lead to an over‐interpretation of the data or a minimisation of the very real structural constraints participants face.

To mitigate these and enhance the trustworthiness of the analysis, I engaged in a continuous process of reflection. This was operationalised through the maintenance of a reflective journal, where I documented emergent assumptions, methodological decisions and analytical dilemmas after each interaction with the data or a participant. During coding and theme development, I sought disconfirming evidence and alternative explanations for the patterns observed. Furthermore, I regularly subjected emerging interpretations to critical questioning, asking how my theoretical predispositions might be shaping the analysis. This iterative reflexive practice was not a separate phase but the foundational ethos of the analytical work, ensuring that the resulting themes, while inevitably shaped by my theoretical lens, remain grounded in the empirical realities.

### 3.7 Ethics

Ethical approval was granted by The Hong Kong Polytechnic University Institutional Review Board. Participants were informed of the study's aims, their right to withdraw without consequence and measures for anonymity. Oral consent was obtained, a procedure approved as appropriate for the study's low‐risk nature and to avoid creating documentation that could potentially breach confidentiality. The ethics committee waived parental consent based on the following considerations: participants were deemed capable of providing independent consent (i.e., able to understand the study and participate voluntarily, with no known serious health or mental capacity issues); were recruited independently of schools via peer networks and the study posed minimal risk. Furthermore, requiring parental consent would have jeopardised participant confidentiality and safety given the sensitive potentially stigmatised nature of the behaviour studied. Pseudonyms (IW01–IW21) were used throughout. To maintain a non‐coercive environment, no nicotine products were ever present. Participants were offered a beverage as a gesture of thanks.

## 4 Findings and Discussion

This section presents an integrated analysis addressing the study's aim: to investigate how adolescent smokers construct a folk sociological imagination to legitimise smoking. Structured around the four research questions, the analysis weaves together empirical findings, theoretical interpretation and relevant literature to elucidate the social processes through which lay epidemiological reasoning reconfigures the public issue of smoking risk into manageable private troubles.

### 4.1 Selective Utilisation of Personal and Peer Observations

Participants privileged firsthand observations and peer‐sourced anecdotes over institutional health information, building an alternative evidence system curated from their immediate social environment. This process, which can be termed anecdotal authority, involved the active curation of a repository of healthy smoker exemplars while disregarding contradictory cases. This system relied on foregrounding specific exemplars whose personal experiences were accorded greater credibility, affirming a core premise of lay epidemiology that individuals privilege knowledge derived from observable socially proximate cases over abstract statistical evidence (Davison et al. 1991; Lovatt et al. 2015).

Diary entries documented a pattern of noting individuals who contradicted narratives of smoking‐induced harm. One participant recorded a common reference point: ‘My friend always talks about his uncle. He's a construction boss, smokes every day but is strong and never gets sick. We use him as proof health warnings aren't always right’ (IW11 Diary, male). Another wrote after a conversation: ‘We were discussing health class and someone mentioned our old Kungfu coach. He smoked but could still perform better than anyone. It made everyone feel the dangers are exaggerated’ (IW07 Diary, male). Within the adolescent context, figures such as the ‘construction boss’ or the ‘Kungfu coach’ thus function as locally credible counternarratives to institutional health messages.

In interviews, participants described how these exemplars became foundational to their reasoning. One participant, when asked about risks, referenced a social figure: ‘There's a popular guy in the grade above who vapes. He's good at basketball and has a lot of friends. You see him and think, if it was really so bad, would he be like that?’ (IW19 Interview, male). Another explained the selective social focus: ‘We don't really bring up people who got sick. It's awkward. It's easier to talk about the ones who are fine, like my cousin. She vapes but is doing well in university. That's the example we remember’ (IW14 Interview, female). This selective attention aligns with broader sociological understandings of motivated reasoning, where culturally or socially valued knowledge is foregrounded to protect practices integral to group identity (Kahan 2013; Kraft et al. 2015).

The selective curation of ‘healthy smokers’ does more than simply neutralise risk; it sustains what Berlant (2011) terms cruel optimism, an attachment to a practice that offers a sense of possibility within an otherwise constrained present. The construction worker who smokes yet remains strong, the university student who vapes yet succeeds. These figures are not merely counterexamples to health warnings; they are vessels for a form of hope that smoking itself makes available, even as that hope is tethered to a practice that forecloses the very futures these adolescents are told to aspire to.

The process emerged as collaborative, with stories being shared and reinforced within groups. One diary entry described an exchange: ‘At lunch, two classmates were talking about a known healthy smoker who lives to an old age. Others joined in agreeing. It's like we gather these cases without even trying’ (IW03 Diary, male). This reveals the process as not merely cognitive but social and agentive. Adolescents gather, validate and amplify these exemplars within peer networks, constructing a shared epistemology that legitimises their practices. This collective curation assembled a body of anecdotal evidence that felt more tangible and trustworthy than abstract warnings, constituting a form of collective sense‐making that manufactures a peer‐validated reality. This curation thereby establishes the foundational pillar of the folk sociological imagination.

### 4.2 Discursive Strategies for Attributing Causality Away From Smoking

Participants engaged in discursive practices that severed the link between nicotine use and health harm, redirecting causality towards a set of externalised or individualised factors. This recalibration of causation, wherein health harms were ascribed to pollution, genetics or individual weakness, served to absolve smoking itself. This attribution insulated their practices from the logical premises of institutional health warnings, illustrating the folk causal component of lay epidemiology (Davison et al. 1991; Lovatt et al. 2015; Pihl et al. 2017).

Diary entries documented the real‐time application of these strategies in peer conversations. One participant recorded an exchange: ‘A friend coughed after vaping and immediately said, “This has nothing to do with the vape; it's just this terrible weather and the air conditioning”. No one questioned it’ (IW09 Diary, male). Another described a group rationalisation about a family member's illness: ‘We were talking about my uncle's lung cancer. Someone said, “It's probably from all the industrial pollution in his city years ago, not from his smoking”. The conversation moved on after that’ (IW06 Diary, male). In these accounts, adverse conditions such as cough were convincingly blamed on environmental factors, demonstrating how vernacular theories of disease aetiology are mobilised to resolve cognitive dissonance between behaviour and risk (Furman 2020; McLaughlin 1996).

In interviews, participants elaborated on these shared logics of causation. When probed about how peers explain health issues in smokers, one stated: ‘The first thing we think is, “What else is wrong with them?” Maybe they have weak genes, or they don't exercise, or they eat poorly. The smoking itself is rarely the main cause we discuss’ (IW20 Interview, male). Another participant framed the issue of dependency through a lens of personal responsibility: ‘Getting addicted is about your own mental strength. If you can't control it, that's on you. The product itself isn't the problem; it's the person's character’ (IW14 Interview, female). This reframing of addiction as a failure of willpower, rather than a chemically engineered outcome, somehow aligns with sociological critiques of healthism, where responsibility for health is individualised and structural factors are minimised (Armstrong 2025; Crawford 2006; Li 2025). These discursive moves also resonate with the concept of ‘techniques of neutralisation’ (Piacentini et al. 2012), through which actors justify deviant behaviours.

By locating harm in pollution, genes or weak will rather than in nicotine itself, participants preserved smoking as a resource for managing the pressures of their daily lives. This illuminates the cruel optimism at the heart of their practice: smoking is clung to not despite its harms but because it offers the readily available technology for manufacturing a sense of control. The attachment is cruel because the object that provides relief from structural constraint also deepens participants' entanglement with it, offering a solution that is also a health trap.

The diaries revealed that these were not isolated opinions but collectively reinforced narratives. One entry noted: ‘Today, someone mentioned a teacher who quit smoking and still got sick. The immediate response was, “See? It's all fate anyway”. It's like we have a ready‐made explanation for every possible argument’ (IW05 Diary, male).

Through these documented conversations, a pattern emerged: physical ailments were routinely ascribed to environmental contaminants or hereditary predisposition, whereas the capacity to quit was framed as a test of individual willpower. This repertoire of causal stories functioned as a communal resource, enabling participants to acknowledge adverse health outcomes while absolving smoking itself of direct blame. Crucially, this study situated these strategies not as mere individual rationalisations but as collectively generated and socially validated discursive resources. The shared nature of these attributions, evident in how peers endorsed explanations, transforms them from private excuses into publicly accepted facts within the group. This collective generation of causal narratives constitutes a second mechanism of the folk sociological imagination, enabling the structural issue of smoking‐related harm to be redefined as a matter of individual circumstance.

### 4.3 Perceived Prevalence as Normative Validation

The perceived ubiquity of nicotine use within participants' immediate social environments functioned as a primary mechanism for validating its acceptability and minimising its perceived risk. This process operates through a form of social proof, where the visible commonality of use is interpreted as de facto evidence of safety. Participants interpreted high visibility among peers and admired figures as such evidence, employing a form of social arithmetic where prevalence correlated with normalcy. This prevalence‐based reasoning is inherent to lay epidemiology, wherein observable local cases are privileged over population‐level statistical evidence (Davison et al. 1991; Lovatt et al. 2015; Pihl et al. 2017).

Diary entries documented observations of widespread use as a central point in risk deliberations. One participant noted, ‘After school, almost everyone at the bubble tea shop was vaping. It felt completely normal. You look around and think, if it was really dangerous, would so many people be doing it?’ (IW02 Diary, male). Another wrote, ‘We saw a group of university students smoking outside their campus. They looked confident and successful. My friend said, “See, everyone does it. It can't be a big deal”’. (IW13 Diary, male). These accounts illustrate how localised norms, observed firsthand, override abstract institutional knowledge.

In interviews, participants articulated how this visible prevalence shaped their perceptions. One stated, ‘When you see your classmates, people you respect, doing it every day without any problems, the school warnings start to feel exaggerated. Your own eyes tell you a different story’ (IW17 Interview, male). Another explained, ‘It's not just about counting people. It's that the people who smoke include the cool students, the sporty ones, the smart ones. If it was harmful, wouldn't the smart ones avoid it?’ (IW21 Interview, male). These perceptions align with social norm theory, which posits that perceived descriptive norms (what others do) powerfully shape behaviour and risk perception (Zhuang and Carey 2025), and hint at dynamics of pluralistic ignorance, where public behaviour is misread as unanimous private approval (Sargent and Newman 2021).

The normalisation process was reinforced through social verification. One diary entry described a reinforcement: ‘Someone expressed worry about health effects, but another friend said, ‘Look how many people smoke in China; do you think all of them will get sick?’ (IW04 Diary, male). This perceived prevalence created a self‐validating social reality where nicotine use became simultaneously normal and normalised. The ‘sheer number’ of observable users, particularly those perceived as successful or admirable, provided participants with continuous, tangible counterevidence to health warnings, transforming statistical risk into a matter of social consensus.

The sight of peers, successful students and admired figures smoking transforms a health‐risk behaviour into a marker of normalcy and belonging. Yet this social proof also illuminates the collective nature of cruel optimism. The group itself becomes the guarantor of the practice's legitimacy, enabling individuals to sustain an attachment that the broader structural environment makes feel necessary. Adolescents are not simply mistaken about risk; they are invested in a shared fiction that smoking can be part of a viable life, because the alternative, facing those pressures without this resource, feels unbearable.

The study revealed how prevalence was not merely perceived but mobilised as an epistemic resource within peer dialogues. Adolescents weaponised visibility, using phrases such as ‘everyone does it’ to rhetorically challenge institutional authority and fortify group consensus. This transformation of observed prevalence into a legitimising discourse constitutes a third mechanism of the folk sociological imagination, whereby the public health issue of population‐level risk is reconfigured into a peer‐validated marker of normalcy and social belonging.

### 4.4 The Folk Sociological Imagination in Practice

The processes of selective exemplification, causal attribution and prevalence‐based normalisation operated collectively to reconfigure the public health issue of smoking risk into a matter of private, individual management. This conceptualisation captures the agentive, communal work through which adolescents dismantle the population‐level logic of public health, reframing risk as a matter of individual constitution. This is achieved through the three interlinked practices detailed above. First, the anecdotal authority of healthy smoker exemplars (Theme 1) provides a tangible counternarrative to statistical warnings. Second, the discursive externalisation of causality (Theme 2) severs the link between nicotine and harm at the individual level. Third, the weaponisation of visible prevalence (Theme 3) transforms common behaviour into a normative safety signal. Together, these practices form a coherent system of vernacular reasoning.

Diary entries revealed how abstract warnings were particularised. One participant wrote after a health class: ‘The teacher showed statistics about cancer rates. We just tuned out. It doesn't feel real. My thinking is: those numbers aren't about me personally; I know my own body and my limits’ (IW12 Diary, male). Another documented a peer's reaction to warning labels: ‘He pointed at the gross picture on the pack and said, “This is just propaganda to scare us. My grandpa smoked the same brand for 40 years and he's fine. It's about the person, not the product”’ (IW19 Diary, male). Here, the logic of anecdotal authority (Theme 1) overrides the population‐level statistic, whereas the personalised attribution of risk to individual constitution (Theme 2) is evident. In these accounts, institutional evidence is not merely disputed but rendered personally irrelevant.

In interviews, participants articulated this reconfiguration more explicitly. One explained: ‘The government talks about “public health” as if we're all the same. But we're not. Whether you get sick depends on your lifestyle, your genes, your environment, not just smoking. So, for me, it's a personal calculation, not a collective danger’ (IW16 Interview, female). Another described their risk assessment framework: ‘I don't think about smoking as the problem. I think: “Can I handle it? Do I have the discipline to control it? Are my genes good?” It becomes about managing yourself, not avoiding a product’ (IW08 Interview, male). This personal calculation integrates all three mechanisms: it relies on self‐exceptionalism (echoing Theme 1), attributes outcomes to individual factors like genes and discipline (Theme 2), and implies a safe norm by treating smoking as a common, manageable behaviour (Theme 3). These personalised frameworks shift responsibility from the public domain of product regulation to the private domain of self‐management.

The social validation of these frameworks was evident in group interactions. One diary entry described, ‘Someone said they were worried about getting addicted. Immediately three friends gave their formulae: “Just exercise more”, “Don't smoke on empty stomach”, “Take vitamins”. We've all developed our own personal strategies instead of quitting’ (IW05 Diary, male). This illustrates how the folk sociological imagination is not an individual cognitive bias but a socially circulated and reinforced toolkit for neutralising risk. The shared generation of these personal formulae demonstrates the collective enactment of the vernacular system synthesised from the preceding themes. These collaboratively forged formulae are more than risk neutralisations; they are vernacular efforts to sustain a sense of control, peer solidarity, and possibility. When participants describe smoking as ‘a break from pressure’, ‘something to look forward to’, or ‘feeling alive’, they articulate what Berlant (2011) identifies as the object of cruel optimism: an attachment that enables one to endure the present because it promises a future, even as that attachment undermines the very flourishing it seems to offer. The folk sociological imagination is thus not merely a cognitive distortion but a socially sustained strategy for manufacturing hope within conditions that afford few others.

This collective sense‐making process dismantled the concept of smoking as a population‐level threat. Participants developed a folk sociological imagination—an agentive vernacular inversion of sense‐making. This operationalises and somewhat extends Mills' (2000) concept. Whereas Mills advocated using the sociological imagination to translate private troubles into public issues, these adolescents enact a folk iteration that inverts this trajectory, translating public health issues into manageable private troubles, complete with personalised mitigation strategies and individualised responsibility frameworks. This inversion is accomplished through the collective practices of peer‐based exemplification, attribution and normalisation. The study's theoretical contribution lies in theorising this process, showing how lay epidemiological reasoning serves as the mechanism for this reconfiguration, and how it is socially generated within peer networks rather than individually held. By foregrounding the collective sense‐making agency of adolescents, the concept of folk sociological imagination challenges the passive subjecthood often implied in social‐environmental models of health behaviour. It offers a nuanced explanation for the persistence of health‐risk behaviours amidst controls, revealing how communities can develop shared epistemic defences that are experientially resonant, socially cohesive and highly resistant to top‐down disconfirmation.

## 5 Conclusions

This study has argued that the persistence of adolescent smoking in China is not merely a failure of policy or a product of social influence but an agentive process of sense‐making. Through the integrated lens of Mills' sociological imagination and lay epidemiology, it demonstrated how adolescent smokers cultivate a folk sociological imagination. This vernacular framework, enacted through peer‐curated exemplification, causal externalisation and the weaponisation of perceived prevalence, reconfigures the public issue of smoking harm into a series of manageable private troubles.

Findings reveal an epistemic gap in current nicotine control. Top‐down messaging likely fails because it cannot compete on the same terrain as the socially validated experience‐based knowledge generated within peer networks. Policies that primarily intensify the volume of discredited messages or strengthen enforcement at point‐of‐sale are therefore destined for limited efficacy, as they leave the collective sense‐making processes that sustain smoking ‘entirely’ untouched. The study also points to an intervention gap in health education, which remains didactic and ill‐equipped to help adolescents deconstruct the lay epidemiological reasoning that circulates in their social worlds. Therefore, there is a need for pedagogical shifts towards ‘participatory methods’ (Strobl et al. 2020) that equip young people to evaluate the very peer‐curated narratives of healthy smokers, externalised causality and safety in numbers that this study has identified. This involves moving from teaching facts to fostering critical health literacy, enabling adolescents to interrogate the sources and social functions of the health beliefs they encounter daily.

Moreover, the study exposes a hope gap that can be understood through Berlant's (2011) cruel optimism. The folk sociological imagination represents a pragmatic, if harmful, response to socio‐structural constraints. Within a pressurised educational system and an uncertain future (Hao et al. 2024; Hou 2024), smoking rituals and their legitimising narratives function as the object of this optimism: they provide crucial, albeit counterproductive, resources for stress management, identity, and belonging (Chen et al. 2022; Hua et al. 2023; Yang et al. 2025). In this light, smoking is not merely a health risk but a culturally available technology for manufacturing immediacy and agency: a way to access moments of camaraderie and autonomy within a tightly structured existence. The attachment is cruel precisely because the smoking practice that sustains a sense of possibility in the present also undermines future flourishing in and beyond health. As the findings demonstrate, this attachment is not passive but actively cultivated through peer‐curated exemplars, causal stories and normalising discourses: a collective investment in a practice that makes the present bearable even as it compromises the future. The challenge for intervention, therefore, is not simply to dismantle this folk epistemology but to recognise the unmet needs it serves.

The study's practical significance is substantial. It moves the problem beyond one of misinformation to one of channelled agency, where the drive for autonomy, belonging and a cruelly optimistic form of hope is directed towards a harmful practice because other avenues are constrained. Consequently, effective intervention must create and legitimise alternative healthier social structures that fulfil the same social and psychological needs adolescents find in smoking. This necessitates a shift in nicotine control strategy, from solely disseminating health warnings towards socially embedded opportunities within schools, communities and peer networks that provide these resources without nicotine use. Ultimately, reducing adolescent smoking depends not just on regulating a product but on engaging with the agentive social worlds of young people.

This study has several limitations that also present avenues for future research. First, the sample was drawn from a highly developed Chinese city using snowball sampling. Although this was methodologically congruent for accessing a hidden population and exploring peer networks, it may limit the transferability of findings to adolescents in rural or less socio‐economically developed regions, where structural constraints and smoking cultures differ. Second, the sample was predominantly male. This facilitated an in‐depth exploration of sense‐making within a key demographic, yet the findings may foreground masculine perspectives and peer dynamics. Given the study's focus on smoking as a resource for identity construction and the exercise of agency, the relative absence of gendered analysis represents a noteworthy omission. Future research could examine how masculinities shape the folk sociological imagination, and whether female adolescents draw upon different symbolic repertoires or encounter distinct social pressures when legitimising nicotine use within more gender‐balanced or female‐dominated peer groups. Third, the focus on smokers and their peer‐validated narratives, although revealing, did not capture the perspectives of non‐smokers or those who have quit, whose insights could provide a valuable counterpoint to the observed lay epidemiological processes. Finally, the analytical focus on shared social meaning required a combined treatment of cigarettes and e‐cigarettes. Whereas this approach was essential for theorising the folk sociological imagination as a unifying process of sense‐making, it necessarily precludes a systematic examination of how rationalisations might differ across product type (e.g., whether distinctive lay theories attach to traditional vs. modern nicotine products).

## Author Contributions

**Bo Li:** conceptualization, methodology, data curation, investigation, formal analysis, writing – original draft, writing – review and editing, validation, software, visualization.

## Funding

This work was funded by The Hong Kong Polytechnic University (Project ID: P0053824).

## Ethics Statement

The Hong Kong Polytechnic University Institutional Review Board ethically approved the research (reference HSEARS20250405001).

## Consent

This study involved no patients; all participants gave oral consent.

## Conflicts of Interest

The author declares no conflicts of interest.

## Permission to Reproduce Material From Other Sources

All content in this article is original and does not include material from other sources.

## Data Availability

Available on request.
